# Evaluating the Effectiveness of Probiotic Supplementation in Combination With Doxycycline for the Treatment of Moderate Acne: A Randomized Double‐Blind Controlled Clinical Trial

**DOI:** 10.1111/jocd.16614

**Published:** 2024-10-16

**Authors:** Najmolsadat Atefi, Masoumeh Mohammadi, Mohammad Bodaghabadi, Marjan Mehrali, Elham Behrangi, Mohammadreza Ghassemi, Alireza Jafarzadeh, Azadeh Goodarzi

**Affiliations:** ^1^ Department of Dermatology, Rasool Akram Medical Complex Clinical Research Development Center (RCRDC), School of Medicine Iran University of Medical Sciences Tehran Iran; ^2^ Department of Geriatric and Gerentology Semnan University of Medical Sciences Semnan Iran; ^3^ School of Medicine Iran University of Medical Sciences Tehran Iran

**Keywords:** acne, acne vulgaris, doxycycline, efficacy, lactobacillus, microbial supplement, microbiome, probiotic, RCT, safety

## Abstract

**Background:**

Acne is a chronic inflammatory skin disease that negatively affects patients' quality of life. Increasing antibiotic resistance is making acne less responsive to treatment. Probiotics are live microorganisms that can provide health benefits by fighting pathogens and maintaining intestinal homeostasis and skin microbiome balance. This study investigates the effects of probiotics in the treatment of acne vulgaris.

**Methods:**

In this randomized controlled clinical trial, 80 patients with moderate acne were divided into two groups of 40. All patients received the same topical treatment, which consisted of a daily antibacterial face wash and Adapalene gel every other night. The control group received one capsule of doxycycline (100 mg) daily, whereas the intervention group received one probiotic capsule daily in addition to doxycycline. Patients underwent photography of facial acne lesions, and treatment response was assessed using the global acne grading system (GAGS) and acne grading method at baseline, as well as during follow‐up visits at 1, 2, and 3 months.

**Results:**

The global acne grading system indicated that both groups showed improvement. However, analyses revealed that outcomes were significantly better in the doxycycline plus probiotics group for the forehead (*p* = 0.018), chin (*p* = 0.021), and nose (*p* = 0.021). No significant differences were observed for the left and right cheeks, back, and chest areas, with the mean GAGS score reduction between the two groups differing by only 2%. Treatment with probiotics significantly reduced the severity of lesions compared to the control group (*p* < 0.001). The acne grading method also indicated that the intervention group had a significantly better treatment response than the control group (*p* < 0.001). Furthermore, treatment with probiotics did not result in any side effects.

**Conclusion:**

Probiotics can serve as an effective and safe treatment option, enhancing the outcomes of routine acne treatments, particularly for patients with acne on the forehead, chin, and nose.

## 1 Introduction

Acne is a prevalent issue for both teenagers and adults, affecting 47%–90% of younger people [1]. Key factors contributing to its development include increased sebum production, hyperkeratinization in pilosebaceous ducts, colonization by *Propionibacterium acnes* (*P. acnes*), and heightened inflammatory responses [2, 3]. Acne is also associated with psychological problems such as anxiety, insomnia, and depression, and can result in dysmorphias and lasting scars if not addressed [4].

The skin contains around one billion bacteria per square centimeter, and imbalances in this microbial community can lead to various non‐infectious conditions, including acne. Treatment approaches vary based on severity and may include local, systemic, phototherapy, or laser therapies [5]. Tetracyclines are effective in inhibiting *P. acnes*, but antibiotic resistance, especially with *Staphylococcus aureus*, is becoming increasingly problematic [6]. Alternative treatments like retinoids and benzoyl peroxide are often used in combination to improve effectiveness but face issues such as side effects and costs [7]. Current recommendations advise against using antibiotics in isolation, highlighting the importance of combination therapies [8].

Probiotics, which are beneficial live microorganisms, have been investigated for their potential benefits in treating various health conditions, including skin disorders. They may aid acne treatment by managing the growth of *P. acnes*, lowering sebum production, and reducing inflammation [9, 10]. Studies suggest that oral probiotics can impact the gut–brain–skin axis, potentially alleviating systemic inflammation and modulating immune responses [11].

This study intends to assess the effects of oral probiotics as a supplementary treatment alongside systemic antibiotics for acne. It represents a new exploration involving a group of 80 participants and will examine how the treatment affects different areas of the face.

## 2 Materials and Methods

**FIGURE 1:**
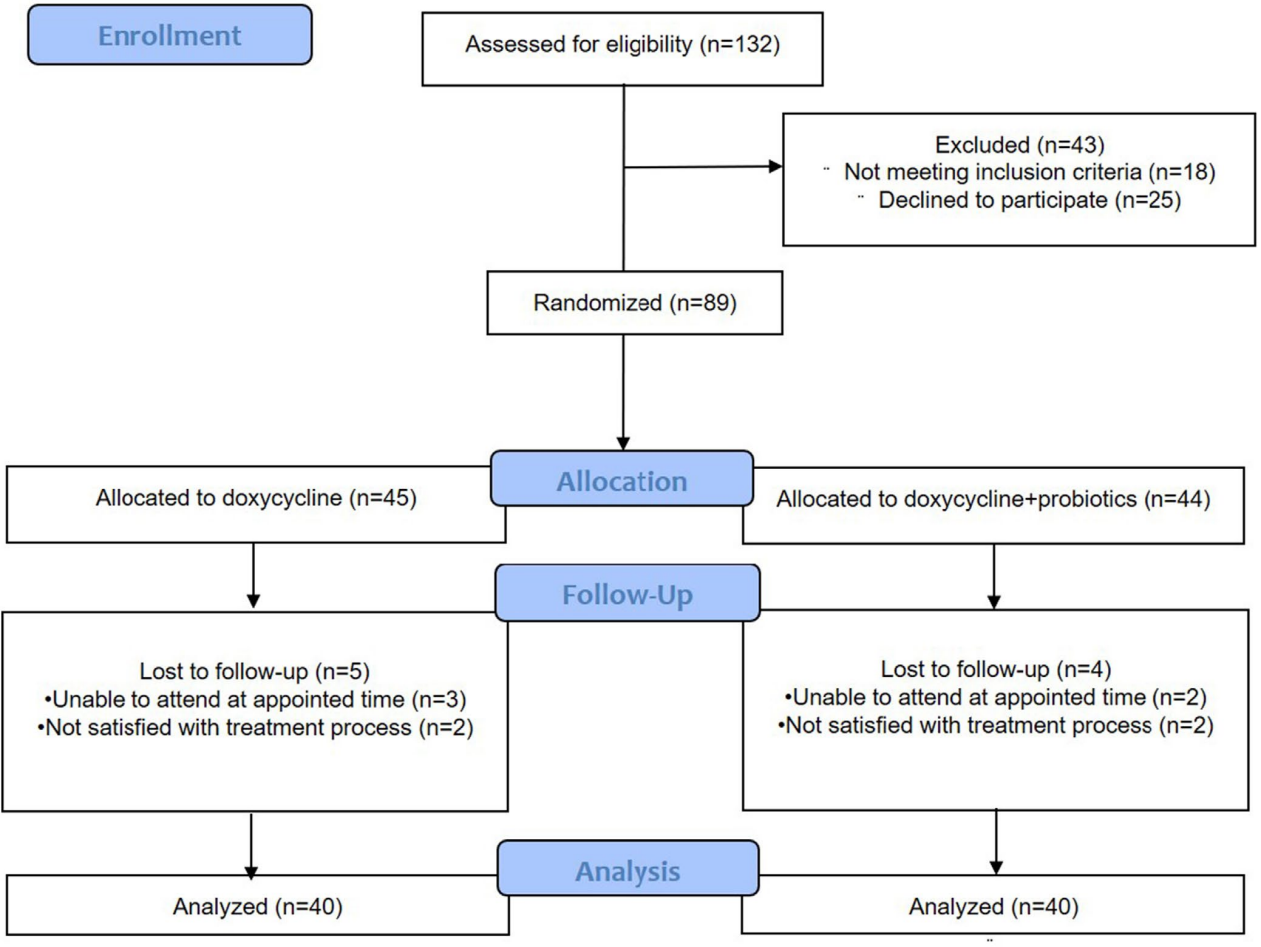
Flow diagram of the phases of the study.

This study was carried out at a healthcare center using a randomized, double‐blind design with two parallel groups, conducted by the researcher and analyst between 2018 and 2020. Considering an alpha level of 0.05 (type I error) and a power of 80% (*β* − 1), as indicated by the GEHAN table and the NW = N/1 − W formula, the estimated sample size for the entire study was determined to be 89 participants (Figure 1).

### 2.1 Eligibility Criteria

Participants aged 15–35 years with moderate acne, defined as grade 2 or 3 on the Global Acne Assessment Scale and exhibiting between 20 and 50 inflammatory lesions, were included in the study. The details of the Global Acne Assessment Scale are found in Table 1.

**TABLE 1:** Global Acne Assessment Scale.

Location	Factor X Grade (0–4) = local score	
Forehead	2	[Global score: 0 = None, 1–18 = Mild, 19–30 = Moderate, 31–38 = Severe, > 39 = very severe]
Right cheek	2	
Left cheek	2	
Nose	1	
Chin	1	
Chest & upper back	3	

Exclusion criteria included pregnant or lactating women, individuals with a history of taking oral probiotics in the past month, those with dyspepsia, and anyone allergic to the study medication.

### 2.2 Random Sequencing and Allocation

Patients were assigned to the treatment groups through random selection in a 1:1 ratio, using a box containing 89 sealed envelopes labeled either A or B. Group A served as the control group, whereas Group B represented the intervention group.

### 2.3 Follow‐Up and Evaluation

In both groups, the response to treatment was assessed using the acne grading method, the Global Acne Grading System (GAGS) [12], Global Acne Assessment Scale (GAAS) [13, 14] and digital photography. Evaluations were conducted at baseline and after 1, 2, and 3 months following treatment. Details of the GAGS and the acne grading method are provided in Tables 3 and 4, respectively.

### 2.4 Blinding

Unlike the first researcher, the second researcher and the analyst only had access to the contractual codes A and B, without knowledge of the actual treatment type for each group.

### 2.5 Therapeutic Regimens

Participants in both groups were subjected to the same topical treatment, consisting of a daily antibacterial face wash and Adapalene cream every other night. Additionally, both groups received oral doxycycline capsules at a dose of 100 mg daily (ENTERIDOX 100 mg capsule, Behshad Darou, Iran) in the evenings to prevent possible photosensitivity. Group B received additional supplementation of two daily probiotic capsules from LactoCare (Zist Takhmir, Iran). Each capsule contains seven probiotic strains: *Lactobacillus casei*, *Lactobacillus acidophilus*, *Lactobacillus rhamnosus*, *Lactobacillus bulgaricus*, *Bifidobacterium breve*, *Bifidobacterium longum*, and *Streptococcus thermophilus*, with a colony count exceeding 10^9^ colony‐forming units.

### 2.6 Statistics and Data Analysis

Statistical analyses were performed using SPSS version 26 software, using the chi‐square test, Fisher's exact test, independent *t*‐test, and logistic regression analysis.

### 2.7 Ethical Approval

This study received approval from the Ethics Committee (registration number: IR.IUMS.FMD.REC.1398.130) and was registered with the Clinical Trial Registration Center (code: IRCT20190830044645N1).

## 3 Results

The present study involved 89 patients with acne, of whom 80 were included in the analyses. Among these, 37 (46.2%) were female and 43 (53.8%) were male. A total of 68 (85%) patients were aged 15–25 years, whereas 12 (15%) were aged 25–35 years. Patients were divided into two groups: those receiving oral probiotics combined with doxycycline and those receiving doxycycline alone. Table 2 presents the demographic characteristics of the two groups, which showed no significant differences (*p* > 0.05).

**TABLE 2:** Demographics of the two groups receiving doxycycline either with or without oral probiotics; *p*‐value, *t*‐test.

Characteristics		Study group		*p*
		Doxycycline + probiotic (*n* = 40)	Doxycycline without probiotic (*n* = 40)	
Sex	Male	20 (50.0%)	23 (57.5%)	0.654
	Female	20 (50.0%)	17 (42.5%)	
Age group	15–25	36 (90.0%)	32 (80.0%)	0.348
	25–35	4 (10.0%)	8 (20.0%)	
Global Acne Assessment Scale	Mild	25 (62.5%)	26 (65.0%)	1.000
	Moderate	15 (37.5%)	14 (35.0%)	
Previous retinoid usage	Yes	36 (90.0%)	39 (97.5%)	0.152
	No	4 (10.0%)	1 (2.5%)	
Hyperandrogenism	No	30 (75.0%)	28 (70.0%)	0.969
	PCO	1 (2.5%)	2 (5.0%)	
	Irregular menstruation	3 (7.5%)	3 (7.5%)	
	Hirsutism	1 (2.5%)	1 (2.5%)	
	Androgenic hair loss	5 (12.5%)	6 (15.0%)	
BMI (mean ± SD)		23.67 ± 2.27	24.11 ± 2.97	0.457

### 3.1 GAGS Scoring Analyses

**FIGURE 2:**
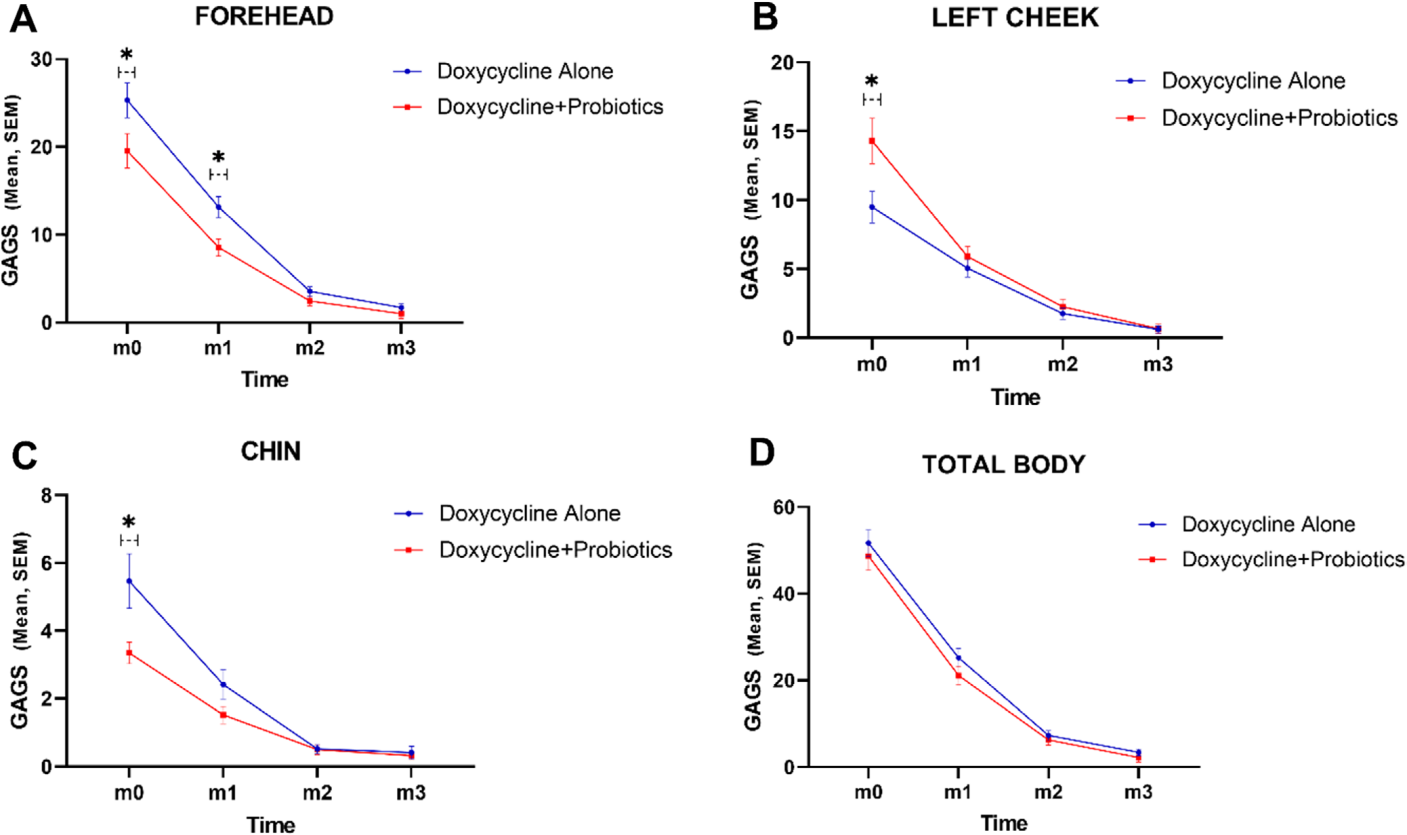
Charts represent mean and standard error of mean (SEM) GAGS score in forehead (A), left cheek (B), chin (C), and total body (D); *p‐value < 0.05.

The mean Global Acne Grading System (GAGS) scores for the forehead, left and right cheeks, chin, nose, back, chest, and total body surface were assessed at four time points, as detailed in the methods section. Table 3 and Figure 2 summarize the statistical analyses concerning the impact of treatment type on each area.

**TABLE 3:** Comparison of mean ± SD GAGS score in different body area between two intervention groups.

	GAGSm0^a^		GAGSm1		GAGSm2		GAGSm3	
	D + P^b^	D	D + P	D	D + P	D	D + P	D
Forehead	19.65 ± 12.32	25.30 ± 12.51	8.55 ± 6.24	13.15 ± 7.45	2.47 ± 3.56	3.57 ± 3.31	1.00 ± 3.32	1.70 ± 2.73
	*p* * = 0.045		*p* = 0.004		*p* = 0.157		*p* = 0.307	
Left cheek	14.30 ± 10.42	9.50 ± 7.17	5.90 ± 4.70	5.05 ± 3.97	2.25 ± 3.57	1.75 ± 2.60	0.65 ± 2.19	0.60 ± 1.64
	*p* = 0.019		*p* = 0.385		*p* = 0.477		*p* = 0.988	
Chin	3.35 ± 2.01	5.47 ± 5.10	1.52 ± 1.58	2.42 ± 2.72	0.5 ± 0.9	0.52 ± 0.81	0.32 ± 0.69	0.42 ± 1.17
	*p* = 0.017		*p* = 0.075		*p* = 0.897		*p* = 0.644	
Nose	0.4 ± 1.44	0.3 ± 0.93	0.12 ± 0.64	0.35 ± 1.44	0.2 ± 0.16	0.0 ± 0.0	0.0 ± 0.0	0.0 ± 0.0
	*p* = 0.715		*p* = 0.371		*p* = 0.324		—	
Back & chest	1.25 ± 4.54	1.65 ± 4.07	0.35 ± 1.91	0.8 ± 2.74	0.0 ± 0.0	0.0 ± 0.0	0.07 ± 0.47	0.0 ± 0.0
	*p* = 0.68		*p* = 0.398		—		*p* = 0.323	
Total body	48.7 ± 20.64	51.72 ± 18.92	21.12 ± 13.49	25.22 ± 13.45	6.28 ± 7.57	7.31 ± 7.11	2.22 ± 6.51	3.4 ± 4.88
	*p* = 0.497		*p* = 0.178		*p* = 0.56		*p* = 0.364	

^a^ GAGS score after 0 month from treatment.

^b^ Doxycycline + Probiotics.

* *p*‐value.

#### 3.1.1 Forehead

The GAGS score for the forehead was significantly higher in the doxycycline‐only group at both baseline and one month (*p* < 0.05). The repeated measures ANOVA indicated a significant effect of treatment type (*p* = 0.018). Additionally, within‐group analysis revealed significant decreases in GAGS scores over time for both groups (*p* < 0.001).

#### 3.1.2 Left‐Side Cheek

The GAGS score was significantly higher at baseline for the Doxycycline + Probiotic group (*p* = 0.019), but no differences were found between groups at later time points (*p* > 0.05). A repeated measures ANOVA showed no significant treatment effect on GAGS scores for the left cheek (*p* = 0.07). However, both groups experienced significant decreases in GAGS scores over time (*p* < 0.001).

#### 3.1.3 Right‐Side Cheek

The repeated measures ANOVA indicated no significant effect of treatment type on GAGS scores for the right cheek (*p* = 0.369). However, within‐group analysis showed significant decreases in GAGS scores over time (*p* < 0.001).

#### 3.1.4 Chin

At baseline, the GAGS score was significantly higher in the doxycycline‐only group (*p* = 0.017), with no significant differences between groups at subsequent time points (*p* > 0.05). The repeated measures ANOVA showed a significant treatment effect on GAGS scores for the chin (*p* = 0.021). Additionally, within‐group analysis revealed significant decreases in GAGS scores over time (*p* < 0.001).

#### 3.1.5 Nose

There were no significant differences in GAGS scores between the two groups at any time point (*p* > 0.05), and the repeated measures ANOVA showed no significant treatment effect for the nose (*p* = 0.907). However, within‐group analysis indicated significant decreases in GAGS scores over time (*p* = 0.021).

#### 3.1.6 Back and Chest

No significant differences in GAGS scores were found between the two groups at any time point (*p* > 0.05), and the repeated measures ANOVA revealed no significant treatment effect for back and chest acne (*p* = 0.574). However, within‐group analysis showed significant decreases in GAGS scores over time (*p* = 0.003).

#### 3.1.7 Total Body

The study assessed GAGS scores for total body acne across four time points for both Doxycycline + Probiotic and Doxycycline‐only groups. Between‐group analysis showed no significant difference in treatment effects (*p* = 0.247). However, both groups exhibited significant decreases in GAGS scores over time (*p* = 0.003). Overall, the treatment types were similarly effective over the four months, with the Doxycycline + Probiotic group showing a marginally greater decrease of only 2% compared to the control group, which was not statistically significant.

### 3.2 GAAS Scoring Analysis

The distribution of Global Acne Assessment Scale (GAAS) classifications in the Doxycycline + Probiotic and Doxycycline without Probiotic groups was not significantly different at all examination times according to the chi‐square test (*p* > 0.05). The rate of GAAS grade reduction was also analyzed using Friedman's test, which indicated a significant decrease in GAAS grade frequency among patients over time (*p* < 0.001). Additionally, Friedman's test revealed a significant main effect of treatment type on GAAS classifications, with the Doxycycline + Probiotic group showing a greater frequency of patients with lower GAAS grades compared to the Doxycycline without Probiotic group.

### 3.3 Acne Grading Method Analysis

The distribution of Acne Grading Method categories in the two groups was analyzed using a chi‐square test, revealing no significant difference between the groups at any examination time (*p* > 0.05). Table 4 presents the distribution of Acne Grading Method categories for the Doxycycline + Probiotic and Doxycycline without Probiotic groups. Friedman's test indicated a significant main effect of treatment type on Acne Grading Method classifications, with the Doxycycline + Probiotic group showing a higher frequency of patients with lower grades compared to the Doxycycline without Probiotic group (*p* < 0.001).

**TABLE 4:** Acne grading method classification distribution in two groups of Doxycycline + Probiotic and Doxycycline without probiotic.

Time		Study group		*p*
		Doxycycline + probiotic	Doxycycline without probiotic	
Acne Grading Method first visit	Grade 2	28 (70.0)	21 (52.5)	0.168
	Grade 4	12 (30.0)	19 (47.5)	
Acne Grading Method month 1, second visit	Grade 0	1 (2.5)	0 (0.0)	0.359
	Grade 2	36 (90.0)	39 (97.5)	
	Grade 4	3 (7.5)	1 (2.5)	
Acne Grading Method month 2, third visit	Grade 0	23 (57.5)	18 (45.0)	0.371
	Grade 2	17 (42.5)	22 (55.0)	
Acne Grading Method month 3, fourth visit	Grade 0	37 (92.5)	33 (82.5)	0.311
	Grade 2	3 (7.5)	7 (17.5)	

*Note:* The table represents frequencies; *p*‐value, chi‐square test.

### 3.4 Side Effects

At the end of the treatment period, side effects were evaluated in both groups. In the doxycycline group, complications included dyspepsia in 9 patients (22.5%), diarrhea in 4 patients (10%), and headache in 2 patients (5%). In contrast, the group receiving combined treatment of doxycycline and probiotics reported dyspepsia in 3 patients (7.5%) and headache in 3 patients (7.5%).

## 4 Discussion

The present study involved 80 patients with acne. An evaluation of the demographic characteristics of the two groups—one receiving oral doxycycline with probiotics and the other receiving doxycycline alone—showed no significant differences.

Statistical analyses demonstrated significant improvements in acne for both groups over the four measurement points. Repeated measures analysis indicated that the addition of probiotics to doxycycline significantly improved acne in the forehead (*p* = 0.018), chin (*p* = 0.021), and nose (*p* = 0.021) compared to doxycycline alone. However, the overall treatment outcomes did not differ significantly between the two groups. The mean reduction in GAGS scores for total body acne at three months was 2% greater in the Doxycycline + Probiotics group.

Acne showed improvement in all areas for both groups over time, with a significant difference observed in the forehead area according to the GAGS criteria. The variation in response rates to the combined treatment of doxycycline and probiotics across different areas can be attributed to factors such as the initial intensity of the lesions, the density of sebaceous glands in the area, and the degree of inflammation of the lesions.

According to the GAAS grading system, the Doxycycline + Probiotics group showed a greater reduction in acne severity compared to the doxycycline alone group (*p* < 0.001). Additionally, statistical analysis using the Acne Grading Method indicated that the intervention group responded better to treatment than the control group (*p* < 0.001).

The most relevant studies conducted worldwide are listed below and compared in Table 5.

**TABLE 5:** A summary of previous studies on the effects of probiotics in active inflammatory acne.

Study	Sample size	Type of study	Probiotics	Adjuvant therapy	Result
Siver et al. [26]	300	Cross‐sectional	*Lactobacillus acidophilus* and *Lactobacillus bulgaricus*	—	Possible interaction between the skin manifestations of acne vulgaris and the metabolic processes of the intestinal tract
Marchetti et al. [27]	40	Clinical trial	*Lactobacillus acidophilus* and *Bifidobacterium bifidum*	Systemic treatment	A better clinical response, wound healing and tolerance to oral antibiotics
Jung et al. [28]	45	Randomized Controlled Trial	*Lactobacillus acidophilus* and *Lactobacillus delbrueckii* subspecies bulgaricus and *Biftdobacterium biftdum*	Minocycline	The response to treatment occurs better and faster in the group receiving probiotics
Kim et al. [29]	36	Randomized Controlled Trial	Lactoferrin	Fermented milk	Decreased sebum content
Muizzuddin et al. [24]	10	Clinical trial	*Lactobacillus plantarum*	—	Reductions in mild acne lesions and erythema plus increases in skin barrier recovery
Kang et al. [23]	70	Randomized Controlled Trial	*Enterococcus faecalis* SL‐5 as a species of *Lactobacillus* genus	—	Inhibition of *P. acnes* and reduction in its inflammatory mediators synthesis. Reduction in inflammatory lesions
Our study	80	Randomized Control Clinical Trial	*Lactobacillus casei, Lactobacillus acidophilus, Lactobacillus rhamnosu, Lactobacillus bulgaricus, Bifidobacterium breve, Bifidobacterium longum, Streptococcus thermophilus*	Doxycycline	No reduction in the whole‐body acne score. Reduction of the inflammatory lesions in the areas of forehead, nose and chin

### 4.1 In Vitro Studies

Laboratory studies have demonstrated the ability of probiotics, such as *Streptococcus salivarius* and *Enterococcus faecalis*, to prevent acne growth through the production of antibacterial proteins. Hacivelioglu et al. [12] found that one such protein, bacteriocin‐like inhibitory substance (BLIS), significantly inhibits the growth of *P. acnes*. Additionally, these probiotics exhibit modulatory effects on the immune system in keratinocytes and epithelial cells, supporting their use as an adjunct in acne treatment.

The beneficial effects of probiotics stem from both direct and indirect mechanisms: they inhibit the growth of *P. acnes*, reduce inflammatory responses, and mitigate the side effects associated with current treatments [13, 14]. Another study highlighted that *S. salivarius*, found in the oropharynx, inhibits *P. acnes* growth in vitro by producing BLIS, suggesting that topical formulations containing BLIS or BLIS‐producing bacteria could be effective for treating acne vulgaris [15].

Moreover, these probiotics inhibit the pro‐inflammatory cytokine IL‐8 in epithelial cells and keratinocytes, leading to immune regulation. This anti‐inflammatory response helps regulate genes related to epithelial adhesion and homeostasis, thus preventing the overgrowth of harmful bacteria without disrupting the skin's microbiome. Probiotics may be particularly beneficial for treating grade 3 and 4 acne, where inflammation is more pronounced [16].

An in vitro study by Wang et al. indicated that skin microorganisms, especially *Staphylococcus epidermidis* (*S. epidermidis*), can ferment glycerol and inhibit the overgrowth of *P. acnes* in culture. Therefore, the presence of *S. epidermidis* may serve as a natural defense against acne, and its increase through probiotic use could improve patient outcomes [17].

Another study reported that bacteriocin produced by Lactobacillus HY499 inhibited inflammation and the growth of skin pathogens, including *S. epidermidis*, *Staphylococcus aureus* (*S. aureus*), *Streptococcus pyogenes*, and *P. acnes*. Notably, bacteriocin did not inhibit the growth of fibroblasts, and no allergic or irritant reactions were observed in human patch tests. The authors explored using bacteriocin from *Lactococcus* sp. HY499 as an antimicrobial agent in cosmetic formulations, emphasizing its safety compared to current treatments [18].

Furthermore, a study conducted by Gueniche et al. showed that *Lactobacillus paracasei* CNCM I‐2116 (ST11) can inhibit skin inflammation caused by substance P and facilitate the regeneration of skin barrier function. ST11 effectively eliminated the effects of substance P, including vasodilation, edema, mast cell degranulation, and TNF‐α release, compared to control samples. The ex vivo skin culture method indicated faster restoration of the skin barrier with ST11, which could be especially beneficial in countering the side effects of traditional treatments that generate free radicals [19].

### 4.2 In Vivo Studies (Topical Probiotics)

Kang et al. [23] investigated the effects of a lotion formulated with Enterococcus faecalis SL‐5 (EFSL5), a species of lactobacilli extracted from human feces, against *P. acnes*. This species shows strong efficacy against gram‐positive bacteria, particularly *P. acnes*. A concentrated powder was prepared from sterilized culture medium, and an aqueous lotion was developed. The placebo consisted of the same lotion but without the CBT SL‐5 powder. Seventy patients diagnosed with mild to moderate acne vulgaris participated in this 8‐week randomized study. Participants were instructed to apply the lotion twice daily to the affected areas. Evaluations were conducted at the beginning of the study and after 2, 4, and 8 weeks. The significant reduction in inflammatory lesions in the intervention group suggests that CBT SL‐5 lotion inhibits *P. acnes* by reducing the production of inflammatory mediators synthesized and released by this pathogen [20].

Muizzuddin et al. observed a reduction in mild acne lesions by diminishing erythema and promoting skin barrier regeneration through a clinical study using *Lactobacillus plantarum* (*L. plantarum*). The formulation containing 5% of *L. plantarum* significantly reduced lesion size and erythema, whereas the 1% formulation did not yield significant results. This study indicates that a formulation with 5% *L. plantarum* may be effective in treating mild acne lesions, and highlights the dose‐dependent effects of lactobacilli, as no benefits were observed with the 1% concentration [21].

### 4.3 Interventional Studies

In 1930, Stokes and Pillsbury first highlighted the therapeutic potential of *Lactobacillus acidophilus* and *L. acidophilus* milk in the inflammatory processes affecting the brain, intestine, and skin. Concurrently, other physicians noted the popularity of *L. acidophilus* cultures among the public as a remedy for acne [22]. However, this earlier research lacked the rigor of clinical trials and merely suggested possible dermatologic benefits.

Despite historical interest, research on probiotic effectiveness remains limited. In 1961, Robert H. Seaver monitored 300 patients using a commercial probiotic (Laxinex tablets containing *L. acidophilus* and *L. bulgaricus*), reporting that 80% of acne sufferers experienced some degree of clinical improvement, particularly those with inflammatory acne. Without a control group, Seaver concluded that “there is an interaction between the skin manifestations of acne vulgaris and the metabolic processes of the intestinal tract” [23]. However, probiotics should not be used as a sole treatment.

In a study involving 40 patients, half received an oral probiotic supplement containing 250 mg of freeze‐dried *Lactobacillus acidophilus* and *Bifidobacterium bifidum* alongside systemic treatment. The probiotic group showed better clinical responses, enhanced wound healing, and improved tolerance to oral antibiotics compared to the control group [24].

Similarly, another study evaluated acne patients with impaired bacterial microflora and found that those receiving gut microbiota‐correcting agents alongside traditional treatments experienced faster clinical improvement than those who did not [25].

In a trial by Jung et al., 45 female patients were divided into three groups: one receiving only probiotics, another receiving probiotics with minocycline, and a third receiving only minocycline. All groups showed significant improvement in lesion counts after four weeks, with continued benefits noted at a 12‐week follow‐up. The combination therapy group experienced a notably greater reduction in lesions, while the minocycline‐only group developed vaginal candidiasis [26]. This study, like ours, demonstrated that the group receiving probiotics had better and faster treatment responses, suggesting that probiotics may serve as a treatment accelerator.

Additionally, a study by Kim et al. involving 36 acne patients indicated that consumption of a lactoferrin‐enriched fermented milk over 12 weeks significantly improved clinical aspects of acne, particularly reducing total lesion counts and sebum production. While lactoferrin (an anti‐inflammatory milk protein) enhanced efficacy, the benefits of probiotics alone further support their role in acne treatment [27].

Currently, numerous studies are examining acne, its severity, and factors influencing treatment effectiveness, including procedural methods for addressing acne and its scars. The authors of this study emphasize ongoing research into the microbiomes associated with acne vulgaris, a common condition and cause of scarring [24, 28, 29].

Further clinical trials involving diverse populations are encouraged, as microbiota composition varies across groups. Our study's sample size may be a limitation, and we urge researchers to investigate the topic more thoroughly, especially concerning severe acne. Future investigations should also consider patients' preferences and adherence to specific probiotic forms. Given the high prevalence of acne, even non‐significant improvements should be acknowledged in clinical practice.

## 5 Limitations

This study has several limitations affecting the interpretation of results. Participant adherence to treatment varied, potentially impacting the effectiveness of doxycycline and probiotics. Additionally, variations in baseline acne severity could skew results, although demographic differences were not significant.

Inconsistencies in follow‐up times may introduce variability in treatment effects, and the sample size of 80 patients limits the generalizability of findings. A larger, multi‐center approach would provide a broader understanding of treatment effects. The short duration of the study (four months) may not capture long‐term effects or recurrence of acne after treatment ends. Lastly, despite using validated scoring systems, potential subjectivity in assessments could affect the outcomes. Addressing these limitations will improve the clarity and reliability of the findings.

## 6 Conclusion

This study demonstrates that both doxycycline with probiotics and doxycycline alone effectively reduce acne, with the combination showing slightly greater improvements in GAGS scores. Clinicians should consider incorporating probiotics into treatment regimens, particularly for patients unresponsive to antibiotics. The findings advocate for personalized treatment approaches in acne management, encouraging clinicians to adopt a holistic strategy that includes adjunctive therapies like probiotics. This could enhance patient outcomes and contribute to evolving standards of care in acne treatment.

## Author Contributions

N.A., Ma.Mo., A.G. and Ma.Me. performed the research. A.J., E.B., M.B. and M.G. designed the research study. A.J. and A.G. contributed essential reagents or tools. Ma.Mo., N.A. and E.B. analyzed the data. M.B. and M.G. wrote the paper. All authors have read and approved the final manuscript.

## Conflicts of Interest

The authors declare no conflicts of interest.

## Data Availability

The data that support the findings of this study are available from the corresponding author upon reasonable request.
